# A Single Acidic Residue Can Guide Binding Site Selection but Does Not Govern QacR Cationic-Drug Affinity

**DOI:** 10.1371/journal.pone.0015974

**Published:** 2011-01-17

**Authors:** Kate M. Peters, Benjamin E. Brooks, Maria A. Schumacher, Ronald A. Skurray, Richard G. Brennan, Melissa H. Brown

**Affiliations:** 1 School of Biological Sciences, University of Sydney, Sydney, New South Wales, Australia,; 2 Department of Biochemistry and Molecular Biology, MD Anderson Cancer Centre Houston, Texas, United States of America; 3 School of Biological Sciences, Flinders University, Adelaide, South Australia, Australia; University of Cambridge, United Kingdom

## Abstract

Structures of the multidrug-binding repressor protein QacR with monovalent and bivalent cationic drugs revealed that the carboxylate side-chains of E90 and E120 were proximal to the positively charged nitrogens of the ligands ethidium, malachite green and rhodamine 6G, and therefore may contribute to drug neutralization and binding affinity. Here, we report structural, biochemical and *in vivo* effects of substituting these glutamate residues. Unexpectedly, substitutions had little impact on ligand affinity or *in vivo* induction capabilities. Structures of QacR(E90Q) and QacR(E120Q) with ethidium or malachite green took similar global conformations that differed significantly from all previously described QacR-drug complexes but still prohibited binding to cognate DNA. Strikingly, the QacR(E90Q)-rhodamine 6G complex revealed two mutually exclusive rhodamine 6G binding sites. Despite multiple structural changes, all drug binding was essentially isoenergetic. Thus, these data strongly suggest that rather than contributing significantly to ligand binding affinity, the role of acidic residues lining the QacR multidrug-binding pocket is primarily to attract and guide cationic drugs to the “best available” positions within the pocket that elicit QacR induction.

## Introduction

The emergence of multidrug resistant pathogens is an ongoing global clinical concern and is significantly driven by the action of multidrug efflux transporters [1], [2]. Capable of exporting a diverse array of structurally and chemically dissimilar drugs from the cell, deciphering the structural mechanisms that govern the polyspecific substrate recognition of these proteins will likely be crucial for the development of future chemotherapeutics.

**Figure 1:**
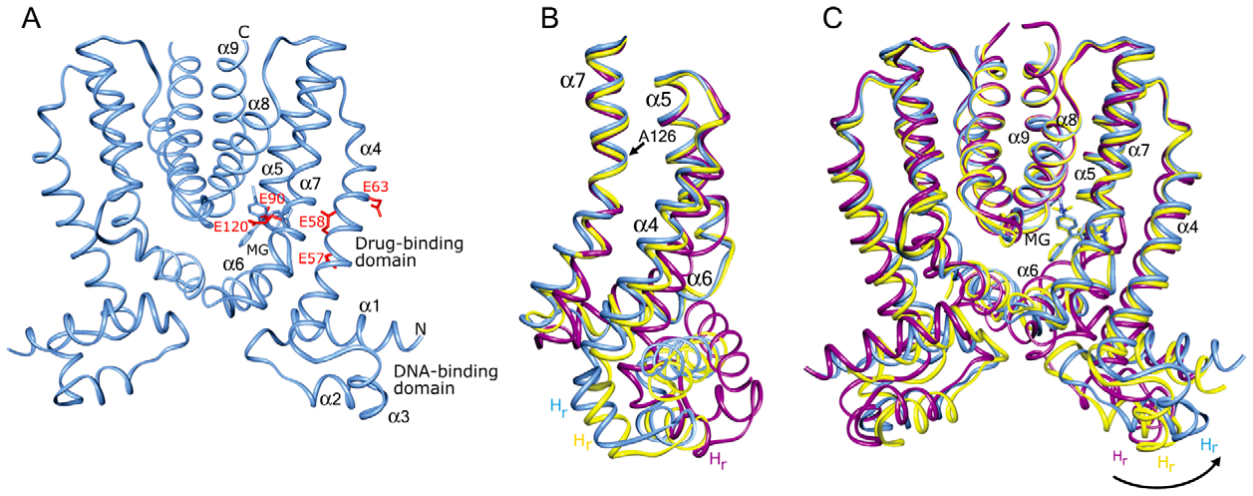
The conformational plasticity of QacR. A) Structure of the wild type QacR-malachite green (MG) complex (space group P4_2_2_1_2). QacR is shown as a light blue ribbon. The nine α helices, N- and C-termini, and DNA- and drug-binding domains are labelled. MG is shown as light blue sticks and the acidic residues of the multidrug-binding pocket, residues E57, E58, E63, E90, and E120, are shown as red sticks. B and C) Superimpositions of one subunit of wild type QacR bound to IR1 DNA (space group P6_5_, magenta ribbon) and MG (space group P4_2_2_1_2, blue ribbon), and QacR(E90Q) bound to MG (space group P6_2_, yellow ribbon) highlighting the pendulum motion of helix α4 (B) and the bending and rotation of helix α7 originating at residue A126 (C) that lead to reorientation of the recognition helices (H_r_) of the DNA-binding domain.

The detailed structural analyses of the *Staphylococcus aureus* multidrug-binding repressor QacR, bound to multiple cationic lipophilic compounds and to two compounds simultaneously [3]–[5], have been influential in affording early insight into the basic principles that govern protein-multidrug interactions. A member of the TetR family of transcriptional regulators [6]–[8], QacR is a dimer composed of nine alpha helices in which helices α1-3 comprise the helix-turn-helix (HTH) containing DNA-binding domain connected via helix α4 to helices α5-α9, which form the dimerization and multidrug-binding domain (Figure 1A).

QacR represses transcription of the *qacA* multidrug efflux transporter gene by binding as a pair of dimers to the IR1 DNA operator sequence, a large inverted repeat that overlaps the *qacA* promoter (P*_qacA_*) [9], [10]. Drug binding occurs with a stoichiometry of one drug per QacR dimer and elicits a coil-to-helix transition of residues T89 through Y93 in the ligand-bound subunit resulting in the expulsion of Y92 and Y93 from the interior of the protein and the formation of the multidrug-binding pocket [3]. This key conformational change also instigates the relocation of the DNA-binding domain, with concomitant smaller changes in the ligand-free subunit resulting in a QacR structure no longer able to bind to the IR1 operator site [3], thus allowing up-regulation of *qacA* transcription.

The QacR multidrug-binding pocket contains two distinct but partially overlapping binding sites designated the rhodamine 6G (R6G) and ethidium (Et) sites after their respectively bound drugs. Key components of this multidrug-binding pocket are five glutamates (Figure 1A), multiple aromatics and several polar and nonpolar residues [3]. Notably, although each cationic drug contacts a distinct subset of QacR residues, all, with the exception of pentamidine (Pt), which is complemented by E63, π-π, cation-π and dipole-charge interactions [5], interact with one or more of the four glutamate residues (E57, E58, E90 and E120) that have been proposed to neutralize their positive charge [3]–[5]. Specifically, E90 interacts with R6G and malachite green (MG) whilst E120 interacts with Et, MG and one of the two positively-charged aminomethylquinolinium moieties of the bivalent drug dequalinium (Dq) [3]. Indeed, many multidrug-binding proteins including; the *Bacillus subtilis* BmrR transcriptional activator [11], [12], the *Escherichia coli* repressor AcrR [13], [14], and the multidrug resistance transport proteins MdfA and EmrE in *Escherichia coli* [15], [16], QacA in *S. aureus* [17], [18] and LmrP in *Lactococcus lactis* [19], predominantly bind cationic substrates with negatively-charged residues featuring prominently in the recognition process. A similar, but inverse importance is found for the regulator TtgR from *Pseudomonas putida* and CmeR from *Campylobacter jejuni*, TetR family members that utilize arginine, lysine or histidine residues for high affinity ligand binding to their anionic inducing compounds [14], [20].

In order to delineate the precise role of the acidic residues within the QacR multidrug-binding pocket in ligand binding and induction, each should be substituted with uncharged side chains, e.g., by alanine or isosterically by glutamine and biochemically and structurally characterized. Surprisingly, such substitutions of residues E57 and E58 caused little change in the binding location or affinity of several drugs with one exception [21]. That exception was the binding of berberine (Be) to QacR(E58Q), whereby the structure revealed a ∼180° rotation of the drug such that new charge neutralization contacts are made to the hydroxyl group of T89 with perhaps some contribution from E90. Intriguingly, this new binding mode increased the affinity of QacR(E58Q) for Be by four-fold. The effects of the removal of the formal negative charges from QacR residues E90 and E120 are unknown. Hence, QacR(E90Q), QacR(E90A), QacR(E120Q), and QacR(E120A) proteins were constructed, purified and tested for their ability to bind to the ligands R6G, MG, Et and Dq. The mutated proteins were analyzed for their ability to bind these drugs both *in vitro* and *in vivo*, and the structures of QacR(E90Q) and QacR(E120Q) in complex with them was determined. The data reveal a striking finding. Similar to residues E57 and E58, glutamine residues at positions 90 and 120 contribute little to cationic multidrug-binding affinity. Rather, in combination with E57, E58 and E63, residues E90 and E120 appear to be responsible for determining the preferred binding location of each individual drug in the multifaceted multidrug-binding pocket and may be utilized to preclude anionic and neutral compounds via their collective negative electrostatic field.

## Results and Discussion

### Drug-binding affinities of the QacR mutants *in vitro*

Drug-binding affinities of wild type (wt), E90 and E120 mutant QacR proteins were determined for MG, R6G and Et utilizing fluorescence quenching of intrinsic tryptophan residues or isothermal titration calorimetry (ITC) for Dq since it displayed significant fluorescence at 340 nm (Figures S1–S5). Unexpectedly, the QacR E90 and E120 mutants displayed only modest effects, no greater than 2.7-fold, on the affinities for the drugs examined (Table 1) indicating clearly that these charged residues are relatively minor contributors to drug-binding affinity.

**Table 1:** Effect of substitutions of QacR residues E90 and E120 on drug-binding affinity (K_d_) *in vitro.*

		Compound (µM)		
QacR protein	Et^a^	Dq^a^	MG^a^	R6G^a^
wt	2.35±0.13	0.90±0.50	1.23±0.14	0.39±0.02
E90A	1.03±0.09	1.80±0.07	1.49±0.10	0.21±0.02
E90Q	1.18±0.04	2.20±0.60	1.74±0.02	0.48±0.05
E120A	1.25±0.15	2.40±0.60	0.98±0.32	0.17±0.01
E120Q	1.16±0.09	2.30±0.50	1.39±0.14	0.58±0.04

a Values represent an average of three separate experiments. Drug binding was measured by tryptophan fluorescence for Et, MG and R6G and by isothermal titration calorimetry for Dq.

### Induction capabilities of the QacR mutants in *S. aureus*

β-lactamase reporter gene assays were used to determine the ability of the QacR mutant proteins to dissociate from IR1 operator DNA in the presence of the inducing compounds Et, Dq, MG and R6G in *S. aureus*. To ensure that NorA, a chromosomally-encoded multidrug resistance transporter that shares a number of substrates with QacA [18], [22], [23] did not mask the ability of compounds to act as inducers of *qacA* transcription, the *S. aureus* strain RN4220 *norA::erm* (SAK1759) [24] was employed. The *blaZ* reporter gene vector pSK5645 [25], and derivatives encoding the wt and mutant QacR proteins, were electroporated into this strain and immunological detection was used to determine mutant protein stability relative to wt QacR protein. The QacR E90 and E120 mutant proteins were present at wt levels (data not shown).

Background β-lactamase activity detected from *S. aureus* SAK1759 strain harboring the pSK5645 plasmid was minimal and was used for normalization. With the exception of QacR E90A, which displayed double the basal induction level, the basal level of expression from P*_qacA_* for the QacR mutants was the same as for wt (Table 2), indicating that DNA-binding was not impaired by these mutations in the QacR multidrug-binding pocket. Inducing compounds were used at concentrations that were below minimum inhibitory concentrations for *S. aureus* containing the large multidrug resistance plasmid pSK1 (data not shown), the natural resource of the *qacA-R* locus, and that afforded maximal induction from P*_qacA_* in the presence of wt QacR. All compounds induced a 2.0- to 3.6-fold increase from the basal level of expression from P*_qacA_* in the presence of wt QacR (Table 2). QacR mutants were also induced in the presence of the drugs examined and displayed similar maximal induction values to those seen with wt QacR in *S. aureus* (Table 2), commensurate with their essentially wt drug-binding affinities *in vitro* (Table 1).

**Table 2:** Effect of substitutions of QacR residues E90 and E120 on induction *in vivo.*

		Compound (µM)							
		Et		Dq		R6G		MG	
QacR protein	Basalinduction^a^	Maximalinduction^a^	x-foldincrease	Maximalinduction^a^	x-foldincrease	Maximalinduction^a^	x-foldincrease	Maximalinduction^a^	x-foldincrease
wt	0.07±0.01	0.14±0.00	2.0	0.22±0.02	3.1	0.17±0.03	2.4	0.24±0.01	3.6
E90A	0.14±0.01	0.19±0.02	1.4	0.23±0.00	1.6	0.17±0.00	1.2	0.24±0.02	1.7
E90Q	0.08±0.01	0.13±0.00	1.6	0.22±0.01	2.8	0.20±0.00	2.5	0.20±0.03	2.5
E120A	0.05±0.01	0.10±0.02	2.0	0.22±0.01	4.4	0.12±0.01	2.4	0.21±0.01	4.2
E120Q	0.07±0.00	0.10±0.00	1.4	0.21±0.01	2.6	0.12±0.03	1.7	0.19±0.00	2.4

a Values, derived from the average of at least two β-lactamase reporter gene assays, are expressed in units, where 1 unit is equivalent to 1 µM of nitrocefin hydrolyzed/min at 37°C. Basal induction was measured in the absence of inducing drug and the concentrations of the QacR ligands required for maximal induction of wt QacR are 17.09 µM Et, 1.90 µM Dq, 1.25 µM MG, and 0.95 µM R6G, respectively.

### Overall structure of the QacR mutant-drug complexes

To understand the lack of significant change in the QacR drug-binding affinities and induction capabilities following substitution of residues E90 and E120, the glutamine-substituted QacR(E90Q) and QacR(E120Q) proteins were crystallized in complex with four structurally dissimilar ligands. Structures of QacR(E90Q) and QacR(E120Q) bound to Et, Dq, MG and R6G were solved to 2.8 Å, 3.3 Å, 2.2 Å and 2.9 Å resolution, or 3.3 Å, 2.9 Å, 2.4 Å and 3.2 Å resolution respectively (Table S1 and Figures S6–S7). The atomic coordinates and structure, factors for the QacR(E90Q)-Dq, QacR(E90Q)-Et, QacR(E90Q)-MG, QacR(E90Q)-R6G, QacR(E120Q)-Dq, QacR(E120Q)-MG and QacR(E120Q)-R6G complexes have been deposited in the Protein Data Bank (http://www.pdb.org) under the accession codes 3br1, 3pm1, 3bqz, 3br5, 3br2, 3br0 and 3br6, respectively. With the exception of Et in complex with QacR(E120Q), wherein no drug density could be clearly ascribed and hence is not discussed further, electron density for each drug was clear and, as observed in previous wt QacR-drug complexes [3], [5], these mutants bound to each drug in a one drug per QacR dimer stoichiometry (data not shown). Structures of QacR(E90Q) and QacR(E120Q) in complex with R6G and Dq crystallized in the same tetragonal P42212 space group as did all of the previously described QacR-drug complexes [3], [5]. Comparison of each of these mutant complexes with the corresponding wt QacR-drug complex gave root mean square deviations (RMSD) of 0.7 Å for all corresponding Cα atoms, indicating no significant global structural changes. By contrast, QacR(E90Q) and QacR(E120Q) in complex with Et and MG crystallized in the hexagonal space group P62 (Table S1).

### A new drug-induced conformation of QacR

Structural comparison of the hexagonal QacR mutant-drug complexes with the corresponding tetragonal wt QacR-drug complex revealed a new QacR conformation (with global RMSDs of ∼2.0 Å between all corresponding Cα atoms) in which notably, residues 89 through 93 had undergone the coil-to-helix transition, the structural hallmark of drug-mediated induction. Comparably few structural differences are noticeable between the individually superimposed DNA-binding domains (RMSDs ≤0.8 Å), containing the HTH region, or multidrug-binding domains (RMSDs ≤1.3 Å) of each QacR subunit, with the most prominent conformational changes apparent in helices α4 and α7 of the multidrug-binding domains which lend to flexibility in the inter-domain orientations.

Superimposition of helices α8 and α9 and their two-fold mates, which together form the dimerization interface and do not move in response to either DNA or drug binding, indicated that the distal ends of helices α4 and α7 of each hexagonal conformer were tilted and rotated in a counter-clockwise direction (CCD) relative to the tetragonal form (Figures 1B–C). Helix α4 moved as a rigid body in a pendulum motion whereas α7 is bent and rotated around residue A126 (Figure 1C). As a direct consequence of its attachment to α4, the DNA-binding domain is rotated in the same CCD by ∼5° from the tetragonal conformer, placing it ∼31° clockwise relative to DNA-bound QacR (Figure 1B). A similar degree of twisting was transmitted in both subunits of the hexagonal dimer. Thus, the hexagonal form of QacR exhibits a closer approach of the DNA-binding domains, and therefore the recognition helices, with their centre-to-centre distance now 39.1 Å compared with 46.4 Å for the tetragonal conformer. This diminished distance is similar to that found between the recognition helices of the DNA-bound form of QacR [3], [10]. However, superimposition of the HTH motifs of QacR(E90Q)-MG onto those of the DNA-bound QacR reveals multiple steric clashes between the hexagonal conformation and the IR1 operator binding site whereby L3 of helix α1 of one subunit is 1.5 Å from the phosphate backbone, the side chain of T24 of helix α2 is 0.7 Å from the phosphate backbone, and the N-termini of the recognition helices clash with DNA bases, e.g., K36 is 2.1 Å and 2.7 Å from the O4 and C7 of one of the thymidines within IR1 (Figure S8). Therefore, the less open but twisted hexagonal QacR conformer is a new, perhaps minimally-induced structure with the more open tetragonal form delimiting the conformational plasticity of drug-bound QacR. Such conformational flexibility also has been observed in the drug-bound form of other TetR family members. Crystal structures of drug-bound EthR and ligand-bound CprB indicated conformational flexibility that allows 5 Å and 2 Å fluctuations, respectively, in the center-to-center distances of the recognition helices of each repressor protein [26], [27]. Such conformational plasticity is also observed for TetR family member AcrR in the absence of multidrug binding, whereby two drug-free structures showed that the recognition helices can flex by at least 3 Å [13], [28], [29].

### QacR mutant-drug interactions: QacR(E90)-Dq and QacR(E120Q)-Dq

**Figure 2 pone-0015974-g002:**
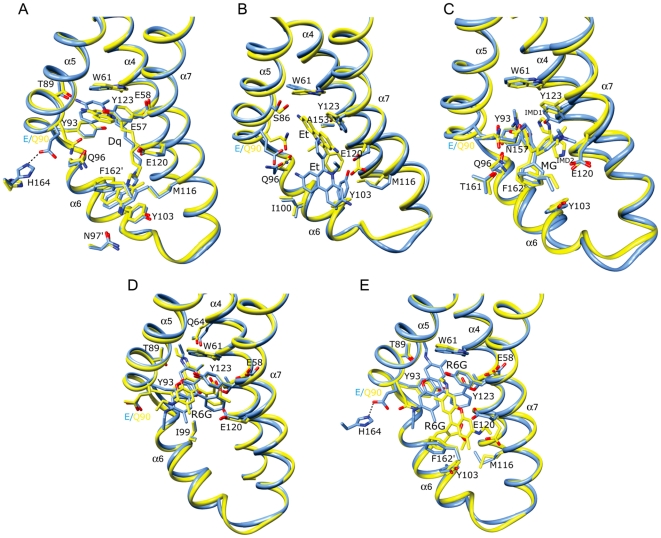
Multidrug binding by QacR(E90Q). A - E) Superimpositions of the structures of the multidrug-binding pockets of wild type (wt) QacR-drug (light blue) and the corresponding QacR(E90Q)-drug (yellow) complexes. For clarity, only the side chains of QacR residues that are within 5 Å of (A) dequalinium (Dq), (B) ethidium (Et), (C) malachite green (MG), (D) rhodamine 6G (R6G) site 1, and (E) rhodamine 6G (R6G) site 2, are shown and labelled. Helices containing drug-interacting residues are labelled in black, and carbon, nitrogen and oxygen atoms are coloured light blue or yellow, blue and red, respectively. The salt bridge between QacR residues E90 and H164 in the wt QacR-Dq and wt-QacR R6G structures is shown as a dashed line in panels A and E. The imidazoles that are found in the drug-binding pocket of the QacR(E90Q)-MG complex are shown and labelled.

**Figure 3 pone-0015974-g003:**
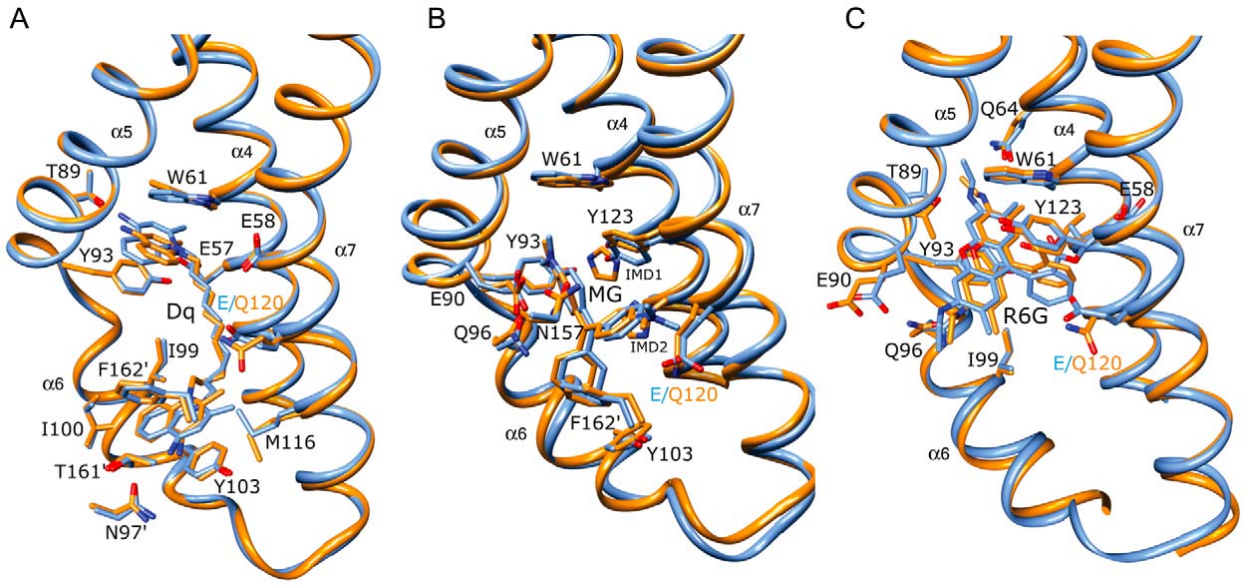
Multidrug binding by QacR(E120Q). Superimpositions of the structures of the multidrug-binding pockets of wild type QacR-drug (light blue) and the corresponding QacR(E120Q)-drug (orange) complexes. For clarity, only the side chains of QacR residues that are within 5 Å of (A) dequalinium (Dq), (B) malachite green (MG), and (C) rhodamine 6G (R6G), are shown. Helices containing drug-interacting residues are labelled in black, and carbon, nitrogen and oxygen atoms are coloured light blue or orange, blue and red, respectively.

The drug-binding site occupied by Dq in the QacR(E90Q)-Dq and QacR(E120Q)-Dq complex structures is nearly identical to that which it takes in the wt QacR-Dq structure and also maintains the majority of protein-drug contacts (Figures 2A and 3A and Table S2). Specifically, Dq spans the length of the multidrug-binding pocket such that one aminomethylquinolinium group remains sandwiched between the aromatic side chains of residues W61 and Y93 in the R6G sub-pocket, and the other between F162′ (where prime indicates the second subunit of the dimer) and Y103 in the Et sub-pocket. Since E90 does not contact Dq in the wt QacR-drug complex, the maintenance of the Dq binding site in the structure of QacR(E90Q)-Dq was not unexpected. Nevertheless, although one of the two positively charged moieties of Dq remained complemented by the carboxylate of residue E120, the E90Q substitution resulted in the loss of a salt bridge between the side chains of H164 and E90. This now untethered glutamine side chain swings to within 3.6 Å of Dq, resulting in the twisting of the proximal aminomethylquinolinium head group by 25° from its wt orientation (Figure 2A). In doing so, there is a 1.5 Å increase in distance between the positively charged N2 nitrogen atom of this Dq moiety and the side chain carboxylate of the “charge-neutralizing” residue E57 (4.8 Å in the wt QacR-Dq complex to 6.2 Å in the QacR(E90Q)-Dq complex). However, alternative, and essentially isoenergetic charge complementation is provided by E58 located 6.1 Å from N2 in the wt QacR-Dq complex and 4.8 Å in the QacR(E90Q)-Dq complex. Consequently, the QacR E90Q substitution is associated with only a moderate and unsurprising 2.4-fold decrease in Dq binding affinity and no impact on induction as observed in the presence of this drug in *S. aureus* (Tables 1 and 2). Indeed, such swapping is analogous to that observed for the mutant proteins QacR(E57Q) and QacR(E58Q) in complex with Dq, wherein residues E57 and E58 afforded immediate functional and chemical redundancy following substitution of either side chain with alanine or glutamine [21].

As observed for QacR(E90Q), the QacR(E120Q) protein retains a near wt level binding affinity for Dq and Dq-induction capability in *S. aureus* (Tables 1 and 2). This retention occurs despite the loss of the “direct” charge complementation of one of the positively charged aminomethylquinolinium groups by the carboxylate of E120 (Figure 3A and Table S2). Intriguingly, no acidic residue is within 6 Å of this positively charged moiety in the QacR(E120Q)-Dq structure. However, the essentially wt *in vitro* and *in vivo* properties of this mutant can be attributed in great part to the retention of the majority of QacR-Dq contacts, including a stronger E57-aminomethylquinolinium interaction, as well as the alternative “neutralization” of the other aminomethylquinolinium moiety by cation-π interactions with the aromatic side chains of Y103 and F162′ and the dipole-charge interaction between the glutamine Oε atom and N1 (Table S2).

### QacR(E90Q)-MG, QacR(E120Q)-MG and QacR(E120Q)-R6G

An essentially wt binding mode is observed for MG in the QacR(E90Q)-MG and QacR(E120Q)-MG structures (Figures 2C and 3B and Table S2). Importantly, the charge of this monovalent cationic drug remained complemented by the unsubstituted carboxylate of either E90 or E120, with the alanine or glutamine substitution of either residue having little impact on binding affinity or induction capabilities of MG (Tables 1 and 2). Analogous to the finding that residues E57 and E58 can functionally substitute for one another in binding Dq [21], residues E90 and E120 appear to provide functional redundancy for MG binding. As expected, the binding site occupied by R6G in the QacR(E120Q)-R6G structure was identical to that in the wt QacR-R6G structure, with the same protein-drug interactions including the charge complementation of this cation by E90 (Figure 3C and Table S2).

Collectively, these data indicate that, in direct contrast to the multidrug-binding proteins EmrE, QacA, LmrP, AcrR and BmrR, for which acidic residues have been shown to provide a direct and significant energetic contribution to ligand binding [11], [12], [14]–[19], residues E90 and E120 are not critical for QacR-cation neutralization or binding affinity. Interestingly, mutational studies with the *E. coli* multidrug exporter MdfA also revealed that although negatively-charged amino acids play a role in substrate recognition and transport their electrostatic interaction is not mandatory for the binding process with cationic substrates [30], [31].

The significant loss of QacR multidrug-binding ability following the seemingly unfavourable mutations of acidic residues is apparently circumvented primarily by new stacking, van der Waals and dipole-drug interactions between different sets of proximal aromatic and polar residues, underscoring the chemical redundancy of the QacR multidrug-binding pocket [3], [21]. Indeed, partial charge complementation of Dq by non-acidic residues in the binding pocket of QacR(E120Q) is comparable to previous observations regarding the neutralization of the bivalent drug Pt by wt QacR [5]. Although in the QacR-Pt complex Pt bound uniquely, by bending into the core of the protein rather than spanning the multidrug-binding pocket like Dq, one of its positively charged benzamidine moieties was neutralized by the carboxylate of E63 and the other, not by acidic residues, but by side chain and carbonyl oxygen atoms contributed by residues S86, Y127, A153 and N157 [5].

One notable new feature of the QacR-MG interaction was revealed by the higher resolution of the QacR(E90Q)-MG and QacR(E120Q)-MG structures (Figures 2B and 3B, and Figures S6C and S7B). Imidazole was observed as two disks of electron density in a pocket between MG and the residues E120 and Y123. The discovery of imidazole in the binding pocket is not surprising as imidazole, which is used in QacR affinity purification and aids in its solubility, is an aromatic cation mimic and thus similar to most of the known QacR ligands. Indeed, four imidazoles were identified in the multidrug-binding pocket of BmrR [12]. The imidazoles bind a site similar to that of the decane linker of Dq in the wt-QacR-Dq, QacR(E90Q)-Dq and QacR(E120Q)-Dq structures (Figures 2A and C and 3A–B).

### QacR(E90Q)-Et and QacR(E90Q)R6G

**Figure 4:**
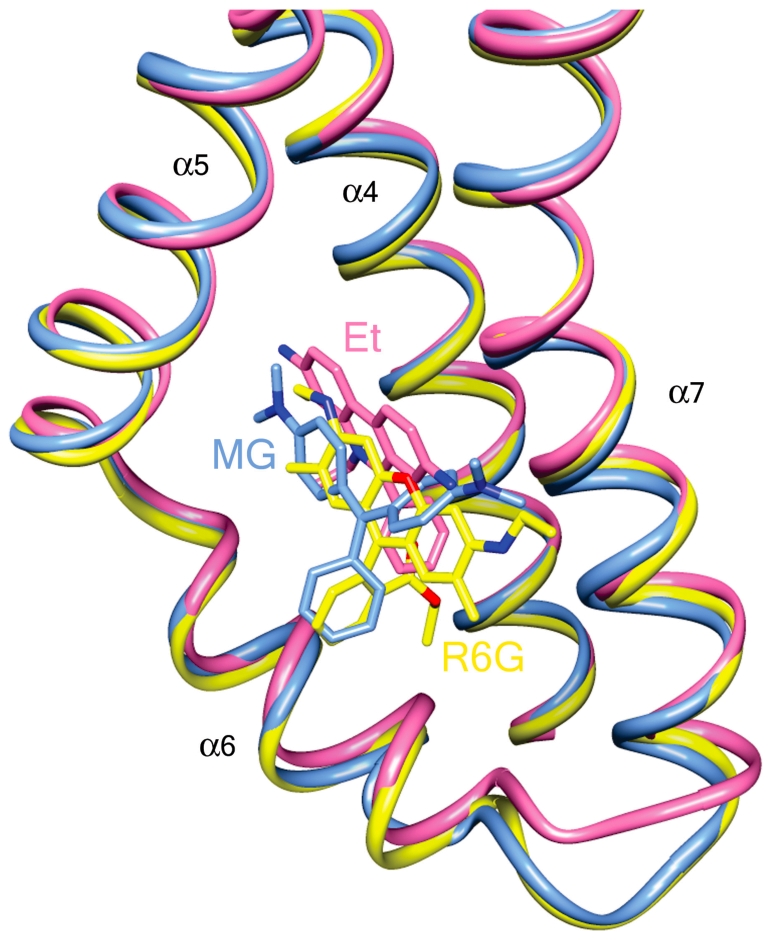
Convergent QacR mutant E90Q and wild type QacR drug-binding sites. Superimposition of the structures of the multidrug-binding pockets of wild type QacR-malachite green (MG; light blue ribbon), QacR(E90Q)-ethidium (Et; pink ribbon), and QacR(E90Q)-rhodamine 6G (R6G; yellow ribbon) structures. Helices containing drug-interacting residues are labelled in black.

Additional intriguing dimensions to the recognized promiscuity of QacR-multidrug interactions are highlighted by the significantly altered binding position of Et in the QacR(E90Q)-Et complex and the two distinct R6G binding sites found in the structure of QacR(E90Q)-R6G. Although charge neutralization of Et was ascribed solely to E120 in the wt QacR-drug complex [3], the location of Et in the QacR(E90Q)-Et structure is dramatically altered (Figures 2B and 4). Indeed, 2F_o_-F_c_ composite omit maps revealed strong and full electron density for Et in this newly revealed binding site despite having relatively high average B-factors (Figure S6B, Table S1). In this QacR(E90Q)-Et complex, Et is angled at 120° relative to its wt orientation and shifted toward the R6G sub-pocket, hence overlapping the Et and R6G sub-pockets and taking a position that is comparable to that occupied by MG (Figure 4). From this location Et now interacts with a new complement of residues distinct from that observed in the wt QacR-Et complex, with a number of drug contacts gained and lost. Lost are van der Waals contacts with the side chains of I99 and I100 and aromatic stacking interactions with F162′ and Y103, the latter residue shifting from a position that would have clashed with Et in the QacR(E90Q)-Et structure. Nevertheless, Et gains stacking interactions with W61 and van der Waals contacts with S86, M116 and N154. Further, the drug maintains contacts with the side chains of residues Q96, E120, Y123 and N157, although the nature of these interactions, in some instances, is altered dramatically. Indeed, in the QacR(E90Q)-Et structure, the side chain of E120, despite remaining within 4 Å of the Et phenanthridinium ring system, is repositioned to avoid a clash with the drug, shifting by 3.0 Å so that it is no longer available to charge complement the Et N5 nitrogen atom (E120-N5 distance  = 4.0 Å in the wt QacR-Et complex and 7.0 Å in the QacR(E90Q)-Et complex). This lost interaction is replaced by a cation-π interaction with Y123 which now stacks with Et. Additionally, the side chain of E90Q swings further into the binding pocket to a position comparable to that observed in the QacR(E90Q)-Dq structure (Figures 2A–B). Thus, relocation of Et in the binding pocket of QacR(E90Q)-Et is apparently, although somewhat enigmatically, driven by the QacR mutation E90Q and subsequent reorientation of the side chains of E90Q, Y103 and E120. Despite the loss of interaction with E90 and employing an almost entirely new complement of QacR residues, this new binding site in the QacR(E90Q)-Et complex exhibited binding affinity and induction capabilities for Et essentially the same as wt QacR (Tables 1 and 2).

In the wt QacR-R6G complex, R6G binds towards the “top” of the multidrug-binding pocket and is complemented electrostatically by E90 [3]. Strikingly, when bound to QacR(E90Q), R6G can now occupy two binding sites (Figures 2D–E). In one site the drug is oriented as observed in the wt QacR-R6G complex (Figure 2D), whereas in the second site the drug is located similarly to Et in the QacR(E90Q)-Et complex, such that R6G (and Et) are bound in a manner comparable to MG, i.e., R6G and Et converge to the more centrally located MG binding site (Figures 2E and 4). The two R6G binding sites are mutually exclusive since, although the coordinate error of the structure indicates that it may be possible to refine R6G into the two binding sites simultaneously, these sites still overlap. Moreover, fluorescence anisotropy binding measurements confirmed that as per wt QacR, QacR(E90Q) binds to R6G in a single drug molecule per QacR dimer stoichiometry (Figure S9). This assertion was further supported by the statistical disorder of the side chains of QacR residues E90Q and E120, each of which appear to have a conformation mutually exclusive with one or the other of the two R6G molecules (Figures 2D–E). In the wt binding site of the QacR(E90Q)-R6G complex, the orientation of the amino acid side chains, including those of E90Q and E120, are identical to those observed in the wt QacR-R6G complex. Nevertheless, in the absence of the side chain carboxylate of E90, the charge complementation of R6G appears to be assumed appreciably by dipole-charge interactions mediated by the side chain oxygen atoms of Q64 and Q96, located within 3.0 and 3.5 Å of the R6G N2 and N1 ethyl amino nitrogen atoms, respectively.

The new R6G binding site is created due to the partial occlusion of the wt site by an alternate conformation adopted by the E90Q side chain, and is also accommodated by a second conformation of the E120 side chain (Figures 2D–E). In this new position, E120 formally complements the R6G N1 nitrogen atom (Table S2). Notably here, R6G also shifts away from contacts with the side chains of residues W61, Q64, T89, Y93, and makes a new van der Waals contact with N157 and new stacking interactions with Y103 and F162′. Strikingly, this binding site is denoted by strong drug density and, also since the R6G binding affinity and induction capabilities of QacR(E90Q) are equivalent to those of wt QacR, this demarks a second physiologically functional and isoenergetic binding site for R6G. Indeed, the thermal parameters of the R6G molecule in this site are somewhat lower than those of the R6G occupying the originally described binding site (Table S1). However, this site appears to exist only in the context of substitution of residue E90.

Thus, QacR(E90Q) contains multiple surfaces employing distinct residue complements capable of isoenergetic Et and R6G binding and induction. Somewhat similarly, the structures of wt QacR in complex with ligands DB75 and DB359 revealed that these bivalent cationic drugs, as ascertained by their weak and missing electron density, bound in multiple orientations within the multidrug-binding pocket [32]. This capacity to bind to the same ligand in multiple orientations also extends to other multidrug-binding proteins, all of which appear to be characterized collectively by voluminous pockets that predominantly employ aromatic stacking interactions for ligand binding. For example, the human pregnane X receptor (PXR) when crystallized in complex with SR12813, one of myriad xenobiotics to which PXR binds, is observed in three distinct binding sites [33]. The *Lactococcus lactis* gene regulator LmrR binds daunomycin in two conformations, both of which utilize stacking with tryptophan residues as an integral part of binding [34]. A single ligand was observed in multiple positions in the multi-ligand binding pocket of the pig odorant binding protein [35]. TtgR binds the plant flavonoid phloretin in high affinity and low affinity sites simultaneously [19], and finally, Pgp has been shown recently to bind a novel cyclic inhibitor (QZ59-SSS) in two locations [36].

Given the subtle impact of the E90Q substitution on the overall structure and, for the most part, on the orientations of the amino acid side chains in the QacR multidrug-binding pocket, it is interesting that these alternate isoenergetic binding sites for Et and R6G have not been observed previously in the wt QacR-drug complexes. The alternate drug-binding sites suggest that the electrostatic contributions of the surrounding acidic residues extend beyond the immediate proximity of each drug observed in the wt QacR. Moreover, the influence of the microenvironment on the formal charge of the glutamates might dictate the preferred location of each drug in the wt protein as well as the shape and local plasticity of the binding pocket. Of course other favourable and unfavourable protein-drug interactions such as π-π and cation-π interactions and steric factors also influence the location of the drug-binding site.

### Conclusion

The similarity between the drug affinities of the mutant and wt QacR proteins for all of the drugs tested here points towards the conclusion that at least for QacR, individual electrostatic interactions between a drug and a proximal “neutralizing” glutamate in the binding pocket are not major contributors to binding affinity. Indeed, a similar lack of effect on drug binding affinity was observed when QacR residues E57 and E58 were substituted with glutamine or alanine [21] and even when double glutamate substitutions (E57Q/E58Q) were made (Peters *et al.*, data not shown). Thus, other protein-drug interactions, most likely aromatic side chain-drug stacking, contribute more significantly to drug-binding affinity. Although formally neutral, aromatic residues offer the ability to interact electrostatically as well as engage in van der Waals interactions. Moreover, inspection of the ligand-binding pockets of both multidrug efflux transport proteins [36]–[39] and other multidrug-binding transcription regulators reveals a plethora of aromatic residues [12], [13], [20], [28]. This is not to suggest that all multidrug-binding proteins do not utilize acidic residues to form strong interactions with their ligands. Indeed, AcrR shows significant effects upon removal of the sole aspartate of the multidrug-binding pocket [13]. However, BmrR exhibits only drug-specific binding defects, whereby R6G binding affinity drops nearly 100-fold in the E253Q substituted protein but Be affinity does not change [12]. Thus, for QacR the formal negative charges of the glutamates may be more important as a guiding mechanism to steer cationic drugs into the binding pocket. Furthermore, their redundancy in QacR allows the loss of anyone of them with little impact on function. Perhaps, such redundancy is common for multidrug-binding proteins that bind both monovalent and bivalent compounds and those proteins, which bind only monovalent compounds, may not display such functional redundancy. An additional nonexclusive role for the binding pocket glutamates of QacR may be to exclude anionic and zwitterionic drugs that could otherwise bind to QacR through charge-charge or charge-dipole repulsion.

Thus, binding site selection of cationic drugs in the QacR multidrug-binding pocket may be driven by a modulated, electrostatic tug-of-war that is in no small part driven by the formal negative charge carried by glutamate residues. Furthermore, these key glutamate residues lining the QacR multidrug-binding pocket may collectively provide a formal negatively charged pathway geared to drive cationic ligands, as much as their structures will sterically allow, toward switch residues T89-T93 at the base of the pocket. This may be a calculated means of ensuring that cationic compounds are adequately inserted to trigger the induction of transcriptional regulators or their translocation mediated by multidrug transporters.

## Materials and Methods

### Bacterial strains and growth conditions

All cloning and over-expression procedures performed in *Escherichia coli* utilized strain DH5α and in *Staphylococcus aureus* strain RN4220 *norA*::*erm* (SAK1759) [24]. Strains were cultured at 37°C in Luria-Bertani broth or agar containing, where appropriate, 100 µg/ml ampicillin for *E. coli* or 20 µg/ml erythromycin and 10 µg/ml chloramphenicol for *S. aureus*. *E. coli* was transformed by standard procedures [40] and *S. aureus* by electroporation as described previously [41], employing a pulse of 1.3 kV.

### DNA isolation and manipulations

The Quantum Prep plasmid miniprep kit (Bio-Rad) and small-scale alkaline lysis procedure [42] were employed to isolate plasmid DNA from *E. coli* and *S. aureus*, respectively. Restriction enzymes, T4 DNA ligase, calf intestinal alkaline phosphatase, Pfu DNA polymerase, and *Taq* DNA polymerase (all from New England Biolabs), were each used according to the manufacturer's instructions. Oligonucleotides were purchased from GeneWorks (Australia) and Invitrogen Corporation (Carlsbad, California). PCR products were purified with the Wizard PCR Prep kit (Promega) and DNA fragments were isolated from agarose gels using the GFX PCR and Gel Band Purification Kit (Amersham Biosciences).

### Construction of QacR mutants

QacR mutants E90A, E90Q, E120A and E120Q were obtained by generating a PCR-derived fragment encompassing the residue to be mutated and replacing the wt sequence as previously described [43]. The K67S substitution, which was found to aid in crystallization, was added to both of the E90Q and E120Q-substituted *qacR* genes using the QuikChange™ mutagenesis protocol (Stratagene). The QacR K67S substitution elicited no observable change in affinity for R6G or IR1 DNA as measured by fluorescence polarization (data not shown). Similar to the C72A/C141S QacR derivative [9], the K67S substitution was thus considered to be a background substitution. Automated DNA sequencing, performed at the Australian Genome Research Facility (Brisbane, Australia), was employed to verify the presence of the desired mutations and the absence of spurious mutations that may have been introduced during PCR.

### Protein preparation

Mutant and wt QacR proteins were over-expressed from *E. coli* cells carrying plasmid derivatives of pSK5676, purified by Ni^2+^-NTA metal chelate affinity chromatography (Invitrogen ProBond resin) as previously described [44], and dialysed against buffer A (1 M NaCl, 20 mM Tris-HCl, 5% [v/v] glycerol, pH 7.5) for tryptophan fluorescence quenching or buffer B (100 mM NaCl, 15 mM Tris-HCl, 2.5% [v/v] glycerol, pH 7.5) for ITC.

### Ligand binding affinity

Tryptophan fluorescence intensity measurements of QacR proteins were performed and K_d_ values calculated as described previously [44]. Each of the reported K_d_ values represents an average of three separate K_d_ determinations. Affinity of QacR proteins for Dq was measured using a VP-ITC microcalorimeter (MicroCal) using protocols as described previously [30].

### Stoichiometry determination

Stoichiometry of QacR(E90Q)-R6G binding was determined by fluorescence polarization using a Beacon 2000 (Invitrogen) fluorescence polarimeter and excitation and emission wavelengths of 490 nm and 560 nm, respectively. R6G was dissolved in buffer B to a final concentration of 1.0 mM and QacR(E90Q) was titrated into the solution up to a final concentration of 5.0 mM.

### Protein stability

QacR protein was detected in whole cell lysates of *S. aureus* SAK1759 containing the desired plasmid using a rabbit polyclonal QacR antibody [9] as previously described [43].

### β-lactamase reporter gene assays

β-lactamase activities of *S. aureus* SAK1759 cells containing the plasmid of interest, were determined using the chromogenic substrate nitrocefin (Oxoid) as described previously [43], [45] and are presented such that 1 unit corresponds to 1 µM nitrocefin hydrolyzed/min at 37°C. Results represent the average of two experiments.

### Crystallization, data collection and structure determination of mutant QacR-drug complexes

Purified QacR mutant proteins were subjected to reductive alkylation of lysines [3], [46], concentrated to 10 mg/ml and buffer exchanged into 50 mM Tris-HCl (pH 7.5), 200 mM NaCl, and 5% glycerol. The resulting protein preparations were incubated with 50–500 µM Et, Dq, MG, or R6G. Crystallization was performed using the hanging drop vapour diffusion method, with each QacR-drug complex mixed 1∶1 with the crystallization solution of 2.3-2.7 M ammonium sulphate and 50–150 mM sodium acetate pH 4.6.

X-ray intensity data from the QacR(E120Q)-R6G, QacR(E120Q)-Dq, QacR(E90Q)-R6G and QacR(E90Q)-Et complex crystals were collected using ALS beam line (BL) 4.2.2; QacR(E90Q)-MG, QacR(E90Q)-Et complex crystals at ALS BL 8.2.2 and the QacR(E90Q)-Dq data at ALS BL 8.2.1 at 100K. Data were processed with MOSFLM and scaled with SCALA [47], [48]. QacR(E90Q) and QacR(E120Q) in complex with R6G and Dq crystallized in the native P4_2_2_1_2 space group whereas crystals of QacR(E90Q) and QacR(E120Q) in complex with Et and MG took the hexagonal space group P6_2_. The structures were solved using MOLREP and the wt QacR structure as a search model (minus solvent and residue 90 or 120) and refined with Refmac5, as implemented in CCP4 [47]. The programs O and Coot were used to visualize and manipulate experimental electron density maps and structures for model building [49], [50]. The stereochemistry of each structure was validated by MolProbity [51] and PROCHECK [52]; PROCHECK was also used for Ramachandran analysis. Table S1 lists selected data collection and refinement statistics for each of the complexes.

Figures 1-4 were prepared with Chimera [53] and Figures S6–S8 were generated in Pymol [54]. Structural alignments were performed in O using the least-squared fitting function (LSQ-EXP/MOL) [50]. The center-to-center distances of the recognition helices (α3) were measured by averaging the positions of the Cα atoms of the residues 37–40 in the middle of each recognition helix and the distance between each of these helical centers determined by the Pythagorean distance formula. Conformational changes were measured with DymDom [55].

## Supporting Information

Figure S1 Stoichiometry of dequalinium binding to wild type QacR. Representative thermogram and data plot is shown. Statistics were compiled from three individual experiments. The binding stoichiometry of greater than one plus the large error reflects errors in the concentrations of protein and drug and their insolubility at higher concentrations.
(TIF)

Figure S2 Stoichiometry of dequalinium binding to the QacR(E90A) mutant. Representative thermogram and data plot showing dequalinium binding to QacR(E90A). Statistics were compiled from five individual experiments. The binding stoichiometry of greater than one plus the large error reflects errors in the concentrations of protein and drug and their insolubility at higher concentrations.
(TIF)

Figure S3 Stoichiometry of dequalinium binding to the QacR(E90Q) mutant. Representative thermogram and data plot is shown. Statistics were compiled from four individual experiments. The binding stoichiometry of greater than one plus the large error reflects errors in the concentrations of protein and drug and their insolubility at higher concentrations.
(TIF)

Figure S4 Stoichiometry of dequalinium binding to the QacR(E120A) mutant. Representative thermogram and data plot is shown. Statistics were compiled from four individual experiments. The binding stoichiometry of greater than one plus the large error reflects errors in the concentrations of protein and drug and their insolubility at higher concentrations.
(TIF)

Figure S5 Stoichiometry of dequalinium binding to the QacR(E120Q) mutant. Representative thermogram and data plot is shown. Statistics were compiled from three individual experiments. The binding stoichiometry of greater than one plus the large error reflects errors in the concentrations of protein and drug and their insolubility at higher concentrations.
(TIF)

Figure S6 2F_o_-F_c_ composite omit electron density maps of QacR(E90Q). In complex with (A) dequalinium, (B) ethidium, (C) malachite green and (D) rhodamine 6G. Molecules are shown as sticks with carbon, nitrogen and oxygen atoms coloured yellow, blue and red, respectively. Two molecules of imidazole are shown in panel C and labelled IMD1 and IMD2. The contour level is 1.0 σ.
(TIF)

Figure S7 2F_o_-F_c_ composite omit electron density maps of QacR(E120Q). In complex with (A) dequalinium, (B) malachite green and (C) rhodamine 6G. Molecules are shown as sticks with carbon, nitrogen and oxygen atoms coloured orange, blue and red, respectively. Two molecules of imidazole are shown in panel B and labelled IMD1 and IMD2. The contour level is 1.0 σ.
(TIF)

Figure S8 Superimposition of the helix-turn-helix motifs of QacR(E90Q)-malachite green and DNA-bound wild type QacR. Overlay of the QacR(E90Q)-malachite green (yellow) and DNA-bound wild type QacR (orange) helix-turn-helix motifs reveals multiple steric clashes between the QacR(E90Q) and the IR1 binding site. Molecules are shown as sticks with nitrogen and oxygen atoms coloured blue and red, respectively. Notably, the β carbon of residue Thr24 (helix α2) is 0.7 Å from the phosphate backbone of thymidine (dT) E24 O3′ and the β carbon of K36 at the N-terminus of the recognition helix is 2.1 Å from the O4 of thymidine (dT) E27 and 2.7 Å from the C7 methyl group.
(TIF)

Figure S9 Stoichiometry of rhodamine 6G binding to the QacR(E190Q) mutant. Representative determination of binding stoichiometry utilizing fluorescence polarization is shown. The value is one drug molecule bound per QacR dimer. All drugs examined with intrinsic fluorescence give the same stoichiometry.
(TIF)

Table S1 Selected data collection and refinement statistics of the eight crystal structures.
(DOC)

Table S2 Distances between residues E57, E58, E90 and E120, and dequalinium, ethidium, malachite green, and rhodamine 6G in the wild type and QacR(E90Q) and QacR(E120Q) mutant-drug complexes.
(DOC)
