# Supplementary figures and images for: A Single Acidic Residue Can Guide Binding Site Selection but Does Not Govern QacR Cationic-Drug Affinity
Source: PLoS One. 2011 Jan 17;6(1):e15974. doi: 10.1371/journal.pone.0015974 (PMC3022030; doi:10.1371/journal.pone.0015974)

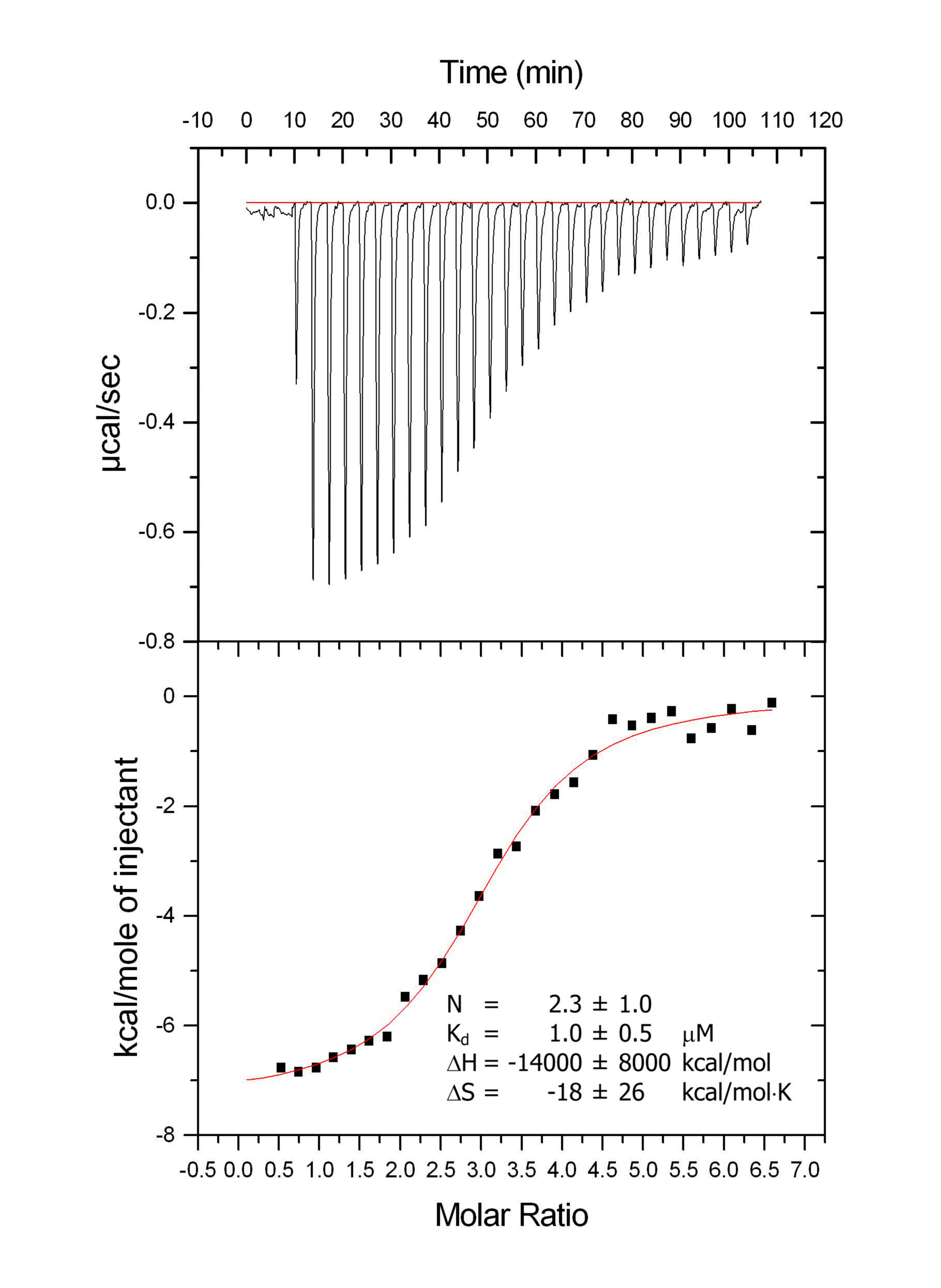

Supplement: Figure S1 — Stoichiometry of dequalinium binding to wild type QacR. Representative thermogram and data plot is shown. Statistics were compiled from three individual experiments. The binding stoichiometry of greater than one plus the large error reflects errors in the concentrations of protein and drug and their insolubility at higher concentrations. (TIF) [file pone.0015974.s001.tif]

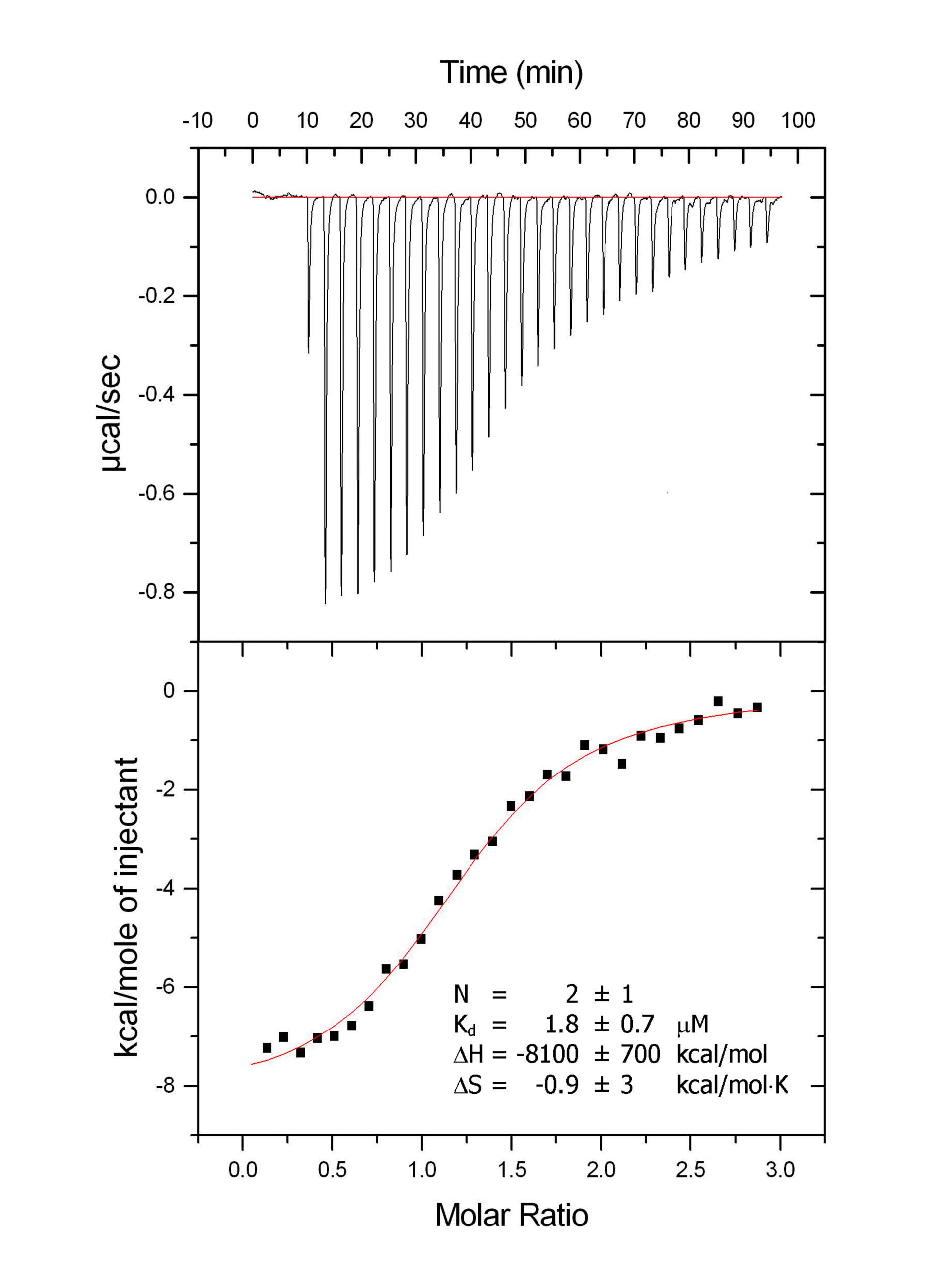

Supplement: Figure S2 — Stoichiometry of dequalinium binding to the QacR(E90A) mutant. Representative thermogram and data plot showing dequalinium binding to QacR(E90A). Statistics were compiled from five individual experiments. The binding stoichiometry of greater than one plus the large error reflects errors in the concentrations of protein and drug and their insolubility at higher concentrations. (TIF) [file pone.0015974.s002.tif]

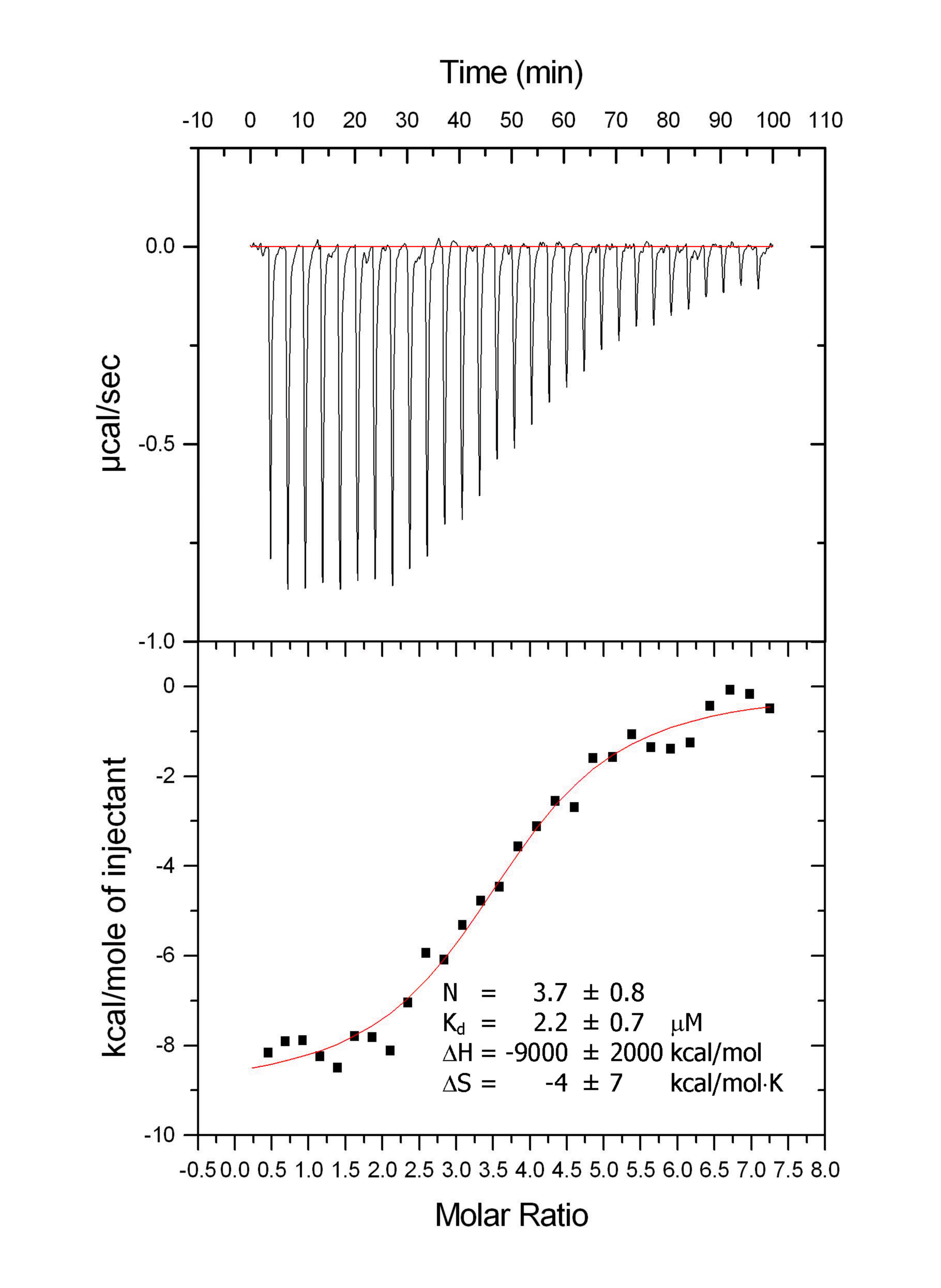

Supplement: Figure S3 — Stoichiometry of dequalinium binding to the QacR(E90Q) mutant. Representative thermogram and data plot is shown. Statistics were compiled from four individual experiments. The binding stoichiometry of greater than one plus the large error reflects errors in the concentrations of protein and drug and their insolubility at higher concentrations. (TIF) [file pone.0015974.s003.tif]

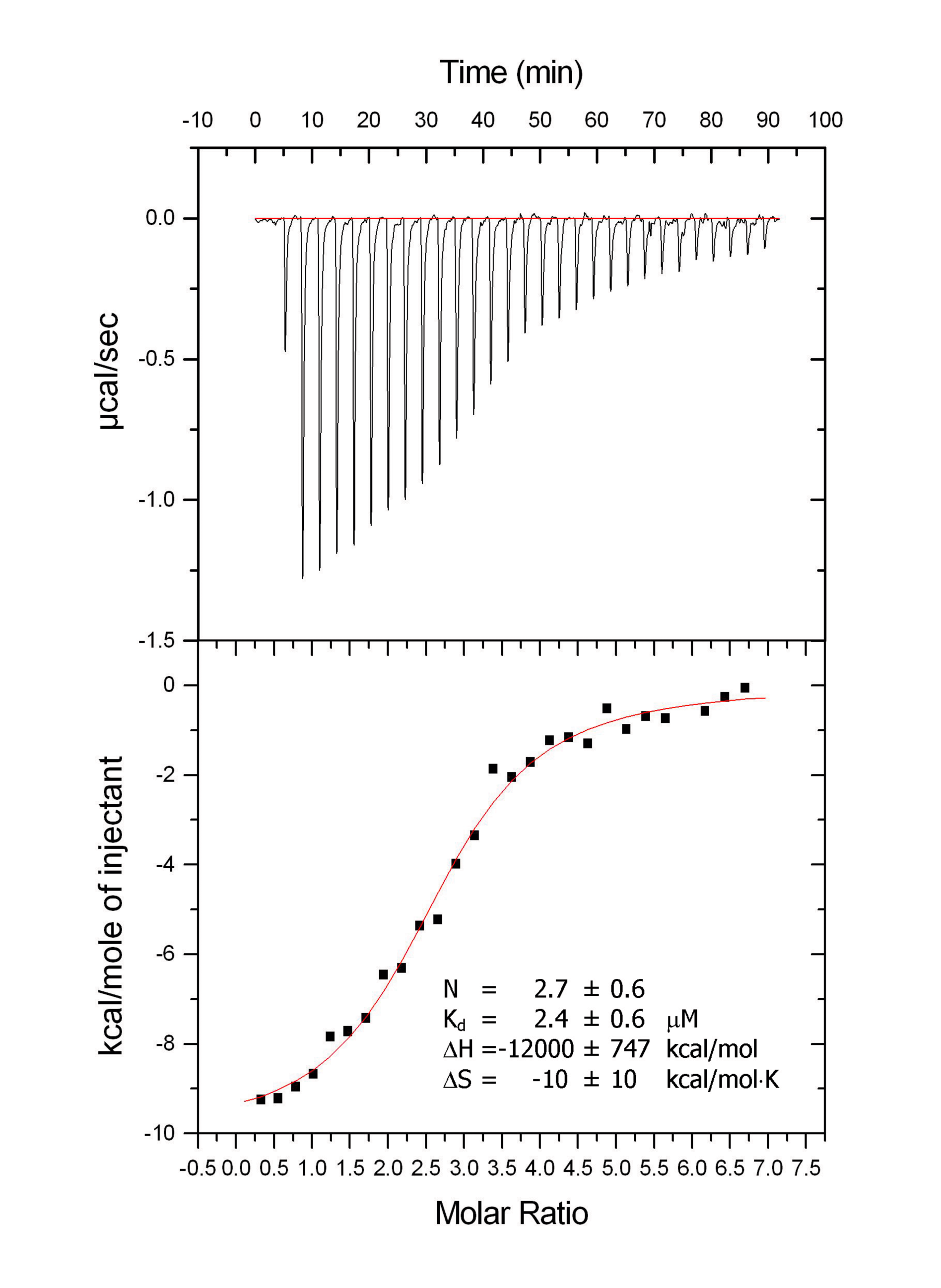

Supplement: Figure S4 — Stoichiometry of dequalinium binding to the QacR(E120A) mutant. Representative thermogram and data plot is shown. Statistics were compiled from four individual experiments. The binding stoichiometry of greater than one plus the large error reflects errors in the concentrations of protein and drug and their insolubility at higher concentrations. (TIF) [file pone.0015974.s004.tif]

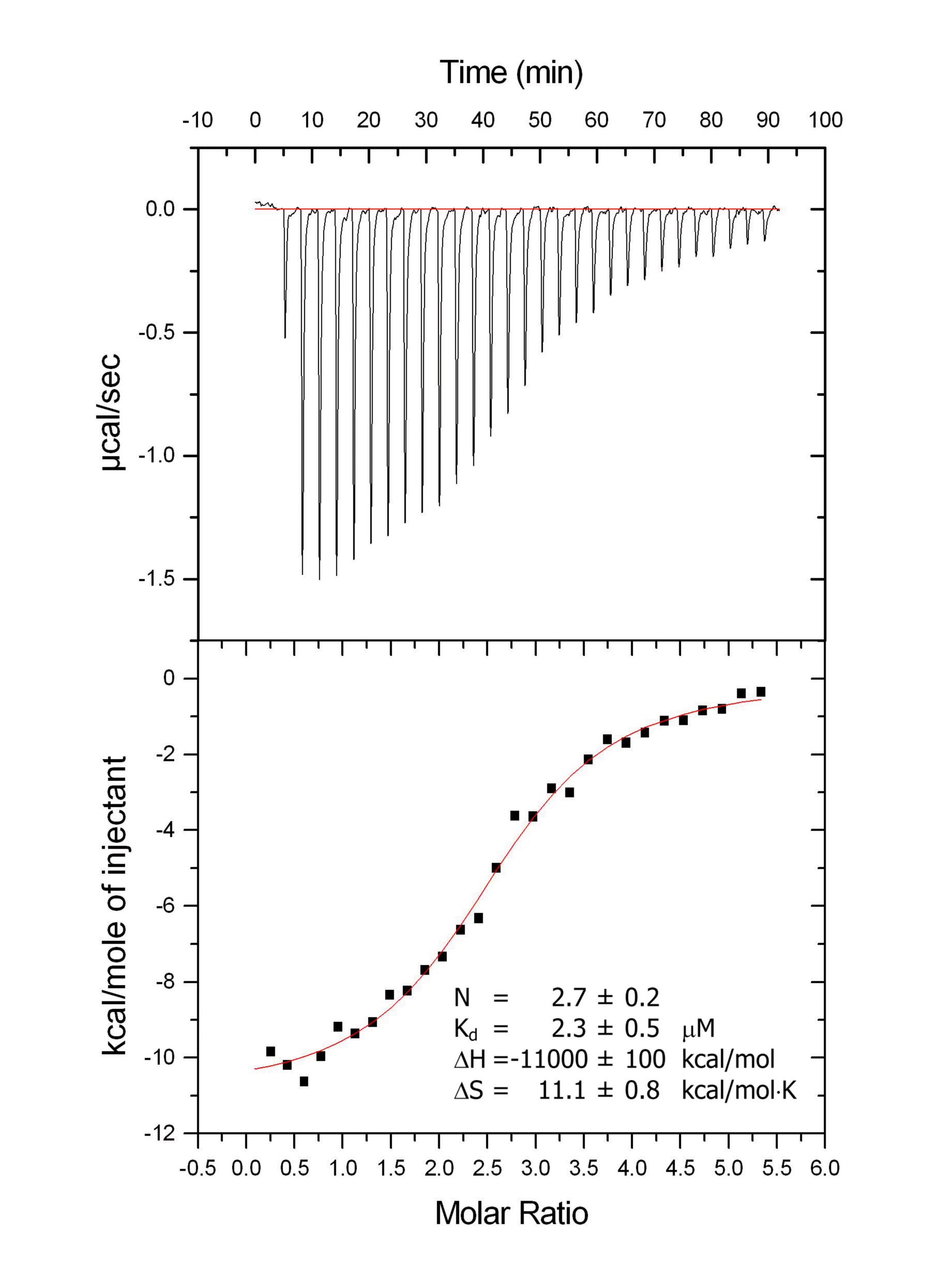

Supplement: Figure S5 — Stoichiometry of dequalinium binding to the QacR(E120Q) mutant. Representative thermogram and data plot is shown. Statistics were compiled from three individual experiments. The binding stoichiometry of greater than one plus the large error reflects errors in the concentrations of protein and drug and their insolubility at higher concentrations. (TIF) [file pone.0015974.s005.tif]

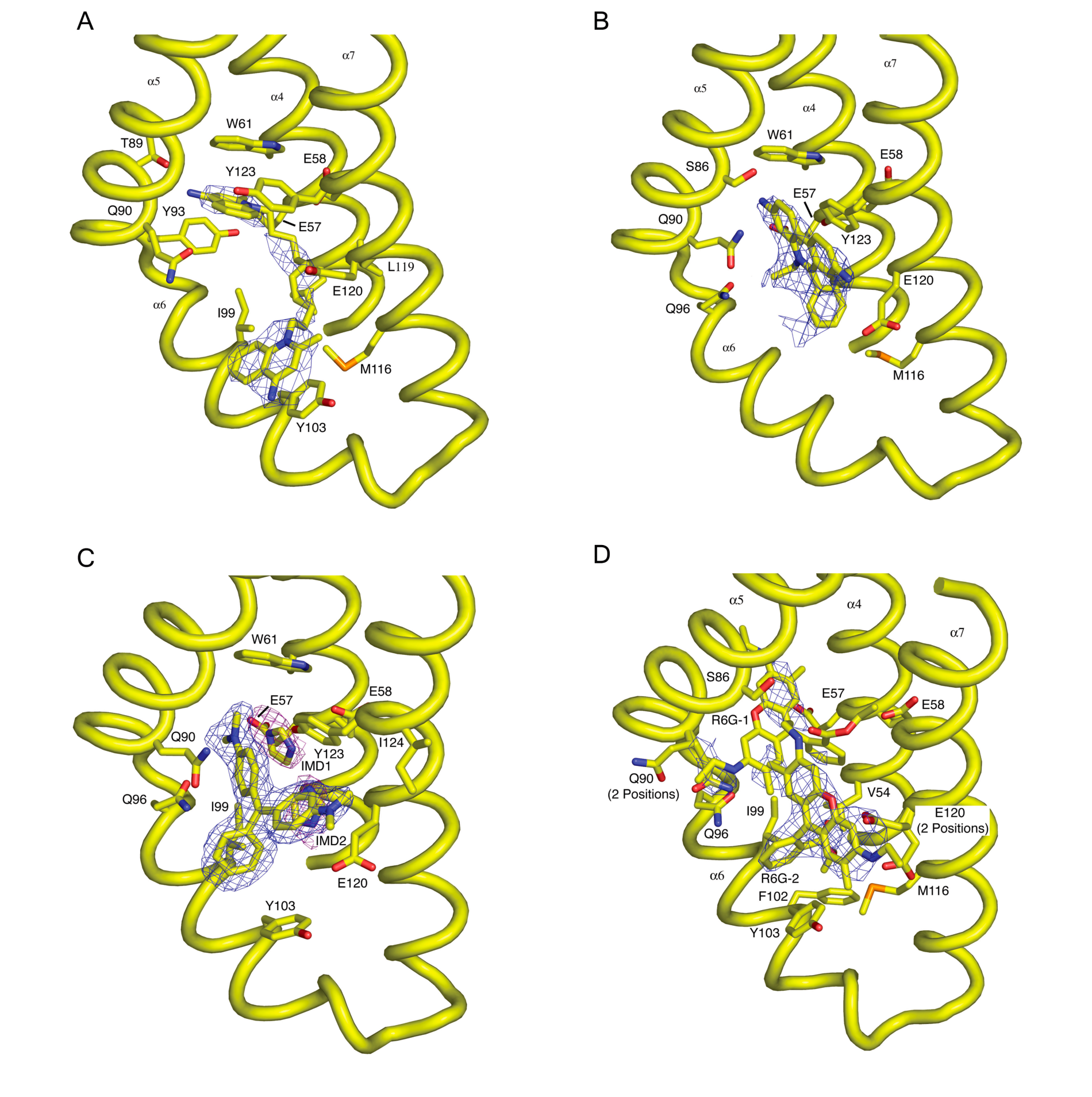

Supplement: Figure S6 — 2Fo-Fc composite omit electron density maps of QacR(E90Q). In complex with (A) dequalinium, (B) ethidium, (C) malachite green and (D) rhodamine 6G. Molecules are shown as sticks with carbon, nitrogen and oxygen atoms coloured yellow, blue and red, respectively. Two molecules of imidazole are shown in panel C and labelled IMD1 and IMD2. The contour level is 1.0 σ. (TIF) [file pone.0015974.s006.tif]

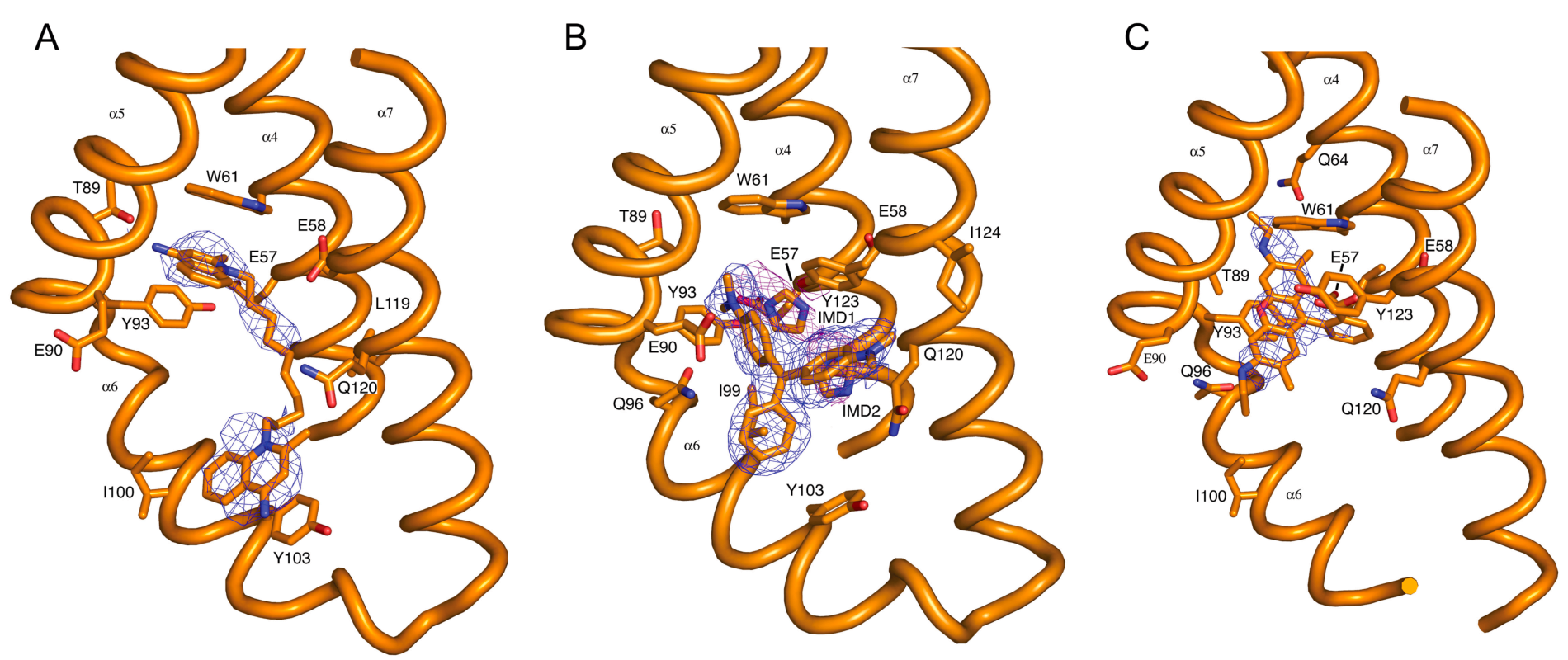

Supplement: Figure S7 — 2Fo-Fc composite omit electron density maps of QacR(E120Q). In complex with (A) dequalinium, (B) malachite green and (C) rhodamine 6G. Molecules are shown as sticks with carbon, nitrogen and oxygen atoms coloured orange, blue and red, respectively. Two molecules of imidazole are shown in panel B and labelled IMD1 and IMD2. The contour level is 1.0 σ. (TIF) [file pone.0015974.s007.tif]

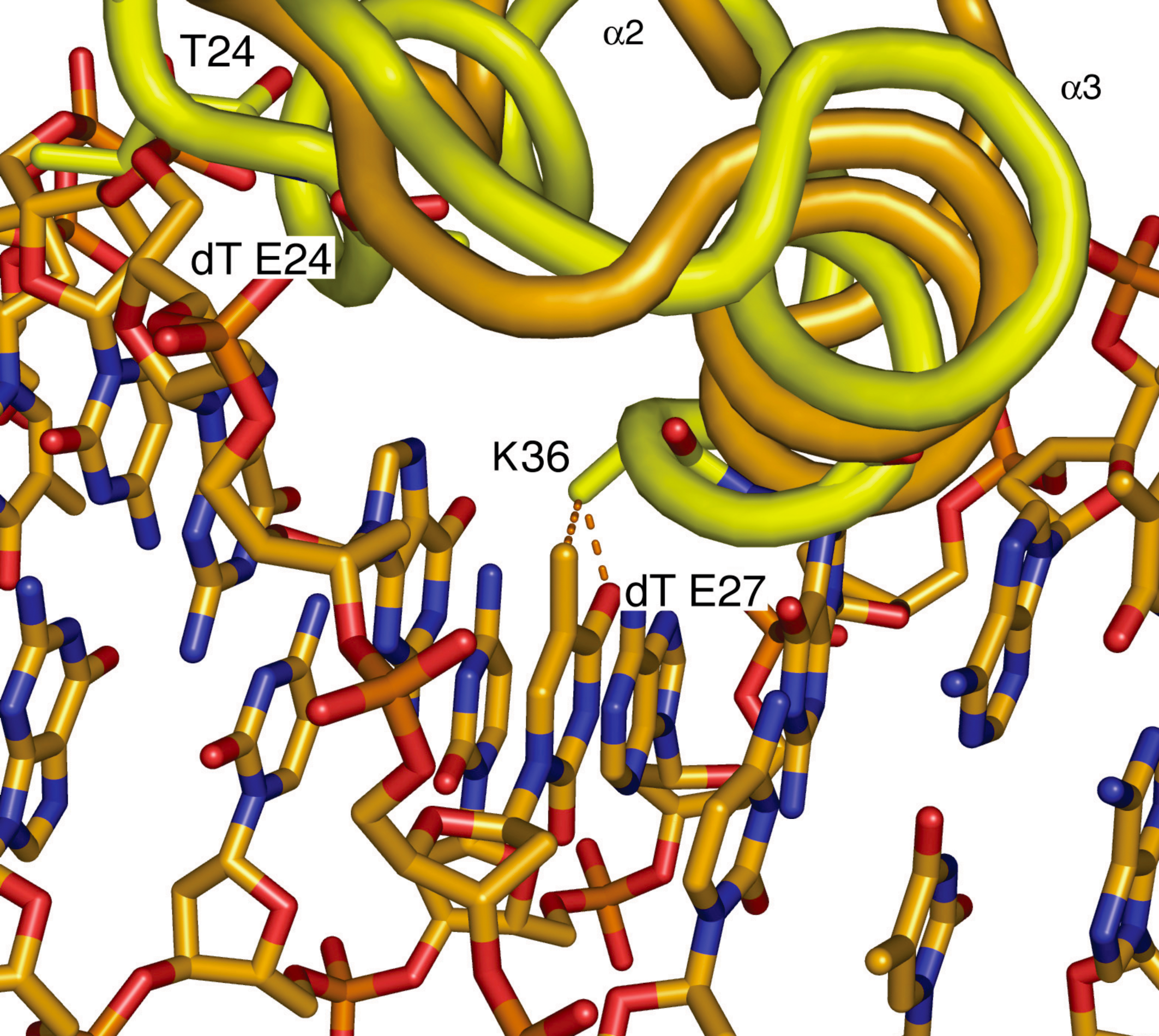

Supplement: Figure S8 — Superimposition of the helix-turn-helix motifs of QacR(E90Q)-malachite green and DNA-bound wild type QacR. Overlay of the QacR(E90Q)-malachite green (yellow) and DNA-bound wild type QacR (orange) helix-turn-helix motifs reveals multiple steric clashes between the QacR(E90Q) and the IR1 binding site. Molecules are shown as sticks with nitrogen and oxygen atoms coloured blue and red, respectively. Notably, the β carbon of residue Thr24 (helix α2) is 0.7 Å from the phosphate backbone of thymidine (dT) E24 O3′ and the β carbon of K36 at the N-terminus of the recognition helix is 2.1 Å from the O4 of thymidine (dT) E27 and 2.7 Å from the C7 methyl group. (TIF) [file pone.0015974.s008.tif]

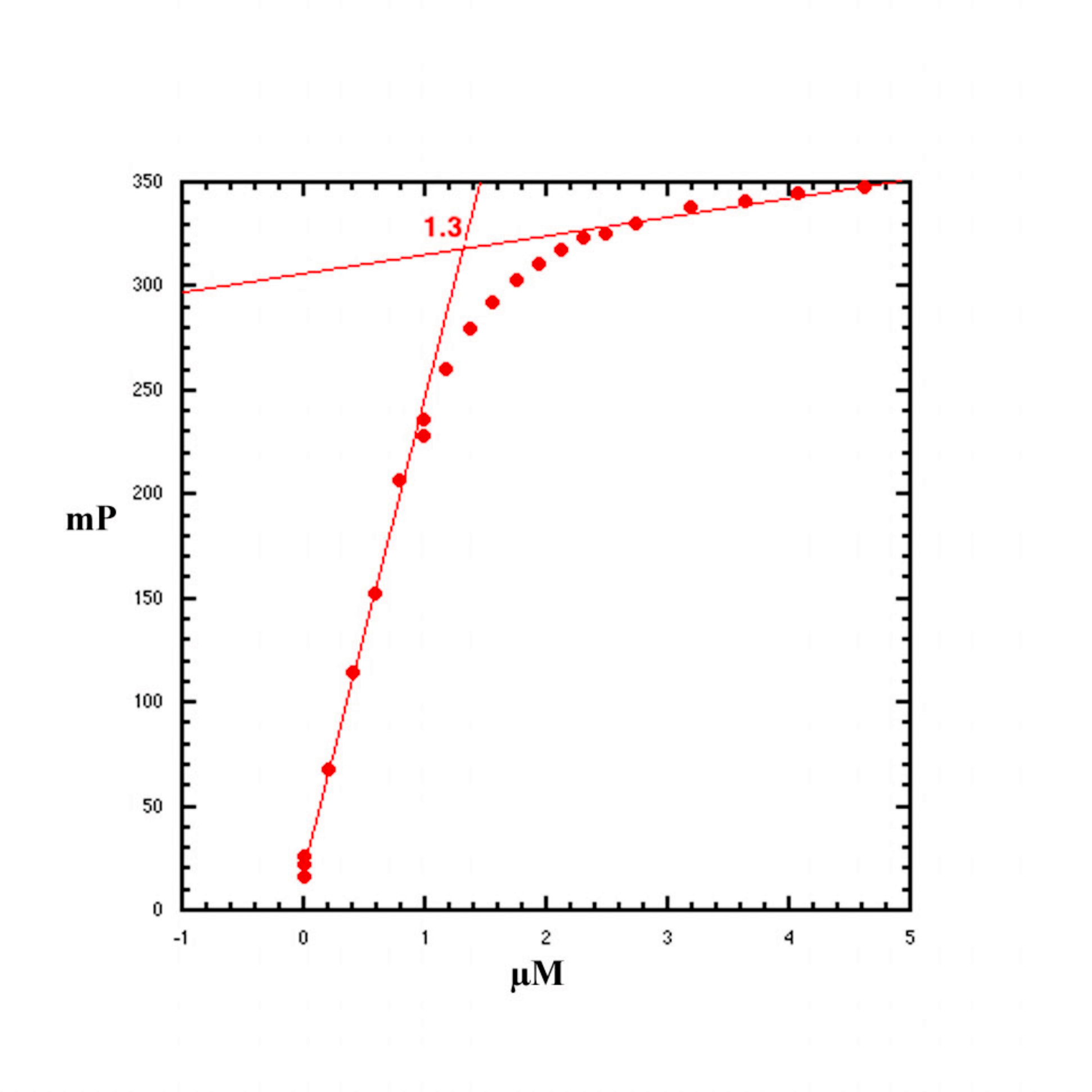

Supplement: Figure S9 — Stoichiometry of rhodamine 6G binding to the QacR(E190Q) mutant. Representative determination of binding stoichiometry utilizing fluorescence polarization is shown. The value is one drug molecule bound per QacR dimer. All drugs examined with intrinsic fluorescence give the same stoichiometry. (TIF) [file pone.0015974.s009.tif]
