# Supplementary material for: A Single Acidic Residue Can Guide Binding Site Selection but Does Not Govern QacR Cationic-Drug Affinity
Source: PLoS One. 2011 Jan 17;6(1):e15974. doi: 10.1371/journal.pone.0015974 (PMC3022030; doi:10.1371/journal.pone.0015974)
Supplement: Table S1 — Selected data collection and refinement statistics of the eight crystal structures. (DOC) [file pone.0015974.s010.doc]

**Table S1. Selected data collection and refinement statistics**

|  | QacR(E90Q)- | QacR(E90Q)- | QacR(E90Q)- | QacR(E90Q)- | QacR(E120Q)- | QacR(E120Q)- | QacR(E120Q)- |
| --- | --- | --- | --- | --- | --- | --- | --- |
|  | Dq | Et | MG | R6G | Dq | MG | R6G |
|  |  |  |  |  |  |  |  |
| Space group | P4_2_2_1_2 | P6_2_ | P6_2_ | P4_2_2_1_2 | P4_2_2_1_2 | P6_2_ | P4_2_2_1_2 |
| Unit cell constants (Å) | a = b = 170.2 | a = b = 104.2 | a = b = 104.9 | a = b = 171.8 | a = b = 171.7 | a = b = 104.2 | a = b = 173.1 |
|  | c = 93.6 | c = 98.6 | c = 93.2 | c = 94.8 | c = 94.4 | c =98.0 | c = 95.9 |
| Resolution range (Å) | 73.9-3.3 | 41.0-2.8 | 45.2-2.2 | 49.0-2.9 | 44.0-2.9 | 35.7-2.4 | 48.0-3.2 |
| Highest resolution shell (Å) | 3.40-3.31 | 2.87-2.80 | 2.23-2.17 | 2.98-2.90 | 2.98-2.90 | 2.48-2.42 | 3.28-3.20 |
| CV-Luzzati coordinate error | 0.5 | 0.4 | 0.2 | 0.4 | 0.4 | 0.2 | 0.5 |
| Overall R_sym_ (%)^a^ | 7.9 (32.4) | 6.1 (40.0) | 6.2 (42.0) | 5.9 (47.9) | 7.1 (52.7) | 8.5 (43.5) | 5.9 (42.4) |
| Overall I/σ(I)^b^ | 8.9 (2.0) | 7.3 (1.9) | 7.4 (1.8) | 7.6 (1.5) | 5.9 (1.5) | 6.1 (1.7) | 6.7 (1.5) |
| Unique reflections (#) | 19,973 | 13,187 | 29,339 | 30,004 | 30,083 | 21,722 | 21,706 |
| Multiplicity | 6.5 | 7.5 | 6.5 | 4.0 | 7.5 | 7.2 | 5.2 |
| Completeness (%) | 100 | 92.4 | 97.0 | 98.8 | 99.7 | 99.2 | 93.3 |
| R_work_/R_free_ (%)^c^ | 21.9/27.8 | 22.9/28.1 | 22.4/25.4 | 24.2/28.4 | 24.7/29.3 | 21.4/26.1 | 22.9/28.3 |
|  |  |  |  |  |  |  |  |
| **Refinement** |  |  |  |  |  |  |  |
| Root mean square deviations |  |  |  |  |  |  |  |
| Bond lengths (Å) | 0.006 | 0.008 | 0.009 | 0.007 | 0.007 | 0.010 | 0.007 |
| Bond angles (deg) | 0.8 | 1.0 | 1.0 | 0.9 | 0.9 | 1.0 | 0.9 |
| B-values (Å^2^) | 0.73 | 1.3 | 1.2 | 0.78 | 0.85 | 1.2 | 0.6 |
| Average B-value (Protein) | 70.8 | 68.0 | 48.3 | 79.2 | 78.7 | 49.3 | 122.5 |
| Average B-value (Drug) | 91.3 | 110.0 | 43.1 | 82.9/61.2^d^ | 117.0 | 40.8 | 99.7 |
|  |  |  |  |  |  |  |  |
| Ramachandran analysis |  |  |  |  |  |  |  |
| Most favored (%) | 92.0 | 91.6 | 95.0 | 91.8 | 91.8 | 95.4 | 92.1 |
| Additional allowed (%) | 6.6 | 6.7 | 3.6 | 7.0 | 6.6 | 3.2 | 6.7 |
| Generously allowed (%) | 0.4 | 0.6 | 0.3 | 0.3 | 0.9 | 0.3 | 0.4 |
| Disallowed (%) | 1.0 | 1.2 | 1.2 | 0.9 | 0.7 | 1.1 | 0.7 |

^a^R_sym_=∑∑|Ihkl-Ihkl(j)|/∑NIhkl, where Ihkl(j) is the observed intensity and Ihkl is the final average value of intensity.

^b^Values in parentheses are the statistics for the highest resolution shell of the data.

^c^R_work_ and R_free_ =∑||F_obs_ - F_calc_||/∑ F_obs_, where F_obs_ = observed structure factor amplitude and F_calc_ = calculated structure factor amplitude for the working and test sets, respectively.

^d^The average thermal parameters of the individual R6G molecules
