# Supplementary material for: A Single Acidic Residue Can Guide Binding Site Selection but Does Not Govern QacR Cationic-Drug Affinity
Source: PLoS One. 2011 Jan 17;6(1):e15974. doi: 10.1371/journal.pone.0015974 (PMC3022030; doi:10.1371/journal.pone.0015974)
Supplement: Table S2 — Distances between residues E57, E58, E90 and E120, and dequalinium, ethidium, malachite green, and rhodamine 6G in the wild type and QacR(E90Q) and QacR(E120Q) mutant-drug complexes. (DOC) [file pone.0015974.s011.doc]

**Table S2. Distances between residues E57, E58, E90 and E120, and dequalinium, ethidium, malachite green, and rhodamine 6G in the wt and QacR(E90Q) and QacR(E120Q) mutant-drug complexes**

| Charge center | QacR protein |  | Distance (Å) |  |  |
| --- | --- | --- | --- | --- | --- |
|  |  | E57 | E58 | E90Q | E120Q |
| Dq^a^ (N1) | wt ^a^ |  |  |  | 4.8 |
|  | E90Q |  |  |  | 3.9 |
|  | E120Q |  |  |  | 3.0 |
| Dq^a^ (N2) | wt | 4.8 | 6.1 |  |  |
|  | E90Q | 6.2 | 4.8 |  |  |
|  | E120Q | 3.2 | 5.1 |  |  |
| Et^a^ (N5) | wt |  |  | 8.2 | 3.9 |
|  | E90Q |  |  | 4.4 | 7.0 |
| MG^a^ (N2) | wt |  |  | 3.4 |  |
|  | E90Q |  |  | 3.5 |  |
|  | E120Q |  |  | 3.2 |  |
| MG^a^ (N3) | wt |  |  |  | 3.6 |
|  | E90Q |  |  |  | 3.7 |
|  | E120Q |  |  |  | 3.5 |
| R6G^a^ (N1) | wt |  |  | 4.0 | 9.0 |
|  | E90Q (Site 1) |  |  | 2.6 | 8.5 |
|  | E90Q (Site 2) |  |  | 10.9 | 2.5 |
|  | E120Q |  |  | 6.4 | 10.5 |

^a^ Dq, dequalinium; Et, ethidium; MG, malachite green; R6G rhodamine 6G; wt, wild type.
